# Early detection of viable *Francisella tularensis* in environmental matrices by culture-based PCR

**DOI:** 10.1186/s12866-020-01748-0

**Published:** 2020-03-25

**Authors:** Helen Y. Buse, Brian J. Morris, Eugene W. Rice

**Affiliations:** 1 US EPA, Office of Research and Development, Center for Environmental Solutions & Emergency Response, 26 W Martin Luther King Drive NG-16, Cincinnati, OH 45268 USA; 2 Pegasus Technical Services, Inc c/o US EPA, Cincinnati, OH USA

**Keywords:** Human pathogen, Potable water, Select agent, Molecular detection, Viability, Remediation

## Abstract

**Background:**

*Francisella tularensis* is a fastidious, Gram-negative coccobacillus and is the causative agent of tularemia. To assess viability yet overcome lengthy incubation periods, a culture-based PCR method was used to detect early growth of the lowest possible number of *F. tularensis* cells. This method utilized a previously developed enhanced *F. tularensis* growth medium and is based on the change in PCR cycle threshold at the start and end of each incubation.

**Results:**

To test method robustness, a virulent Type A1 (Schu4) and B (IN99) strain and the avirulent Live Vaccine Strain (LVS) were incubated with inactivated target cells, humic acid, drinking and well water, and test dust at targeted starting concentrations of 1, 10, and 100 CFU mL^− 1^ (low, mid, and high, respectively). After 48 h, LVS growth was detected at all targeted concentrations in the presence of 10^6^ inactivated LVS cells; while Schu4 and IN99 growth was detected in the presence of 10^4^ Schu4 or IN99 inactivated cells at the mid and high targets. Early detection of *F. tularensis* growth was strain and concentration dependent in the presence of fast-growing well water and test dust organisms. In contrast, growth was detected at each targeted concentration by 24 h in humic acid and drinking water for all strains.

**Conclusions:**

Results indicated that the culture-based PCR assay is quick, sensitive, and specific while still utilizing growth as a measure of pathogen viability. This method can circumvent lengthy incubations required for *Francisella* identification, especially when swift answers are needed during epidemiological investigations, remediation efforts, and decontamination verification.

## Background

*Francisella tularensis* is the causative agent of tularemia, a disease with several clinical manifestations depending on the transmission route [1]. Tularemia can result from the inhalation of contaminated dust and aerosols, bites by infected vectors, contact with infected animals, or ingestion of contaminated food and water [reviewed in [2]]. Although the environmental reservoir for *F. tularensis* is unclear, it has been found within rodents, lagomorphs, and arthropods [3, 4], as well as in numerous aquatic environments, e.g. surface water and sediments, brackish water, and other open water sources [5]. Contaminated surface, well, and domestic rural water, as well as community water supplies with unchlorinated or inadequate treatment processes, have all been implicated as the sources of *Francisella* outbreaks [reviewed in [6]] and suggests that persistence within aquatic environments may be important in *Francisella* ecology.

*F. tularensis* is divided into three subspecies which vary in their pathogenicity and geographic distribution: *tularensis* (Type A), *holarctica* (Type B), and *mediasiatica* with ongoing debate whether to classify *F. novicida*, as either a fourth subspecies or separate species [7, 8]. Type A strains are further divided into two subpopulations, A1 (A.I. or A-east) and A2 (A.II. or A-west) with four distinct genotypes (A1a, A1b, A2a, and A2b) [9]. In the U.S., tularemia can be caused by both Type A and B strains with higher rates of mortality resulting from infections with A1b strains compared to A1a, A2, and Type B strains [9, 10].

Due to its low infectious dose, high virulence, and transmissibility by air, misuse of *F. tularensis* has the capability to cause a high-consequence event with potentially large casualties, negative effects on the economy and infrastructure, and threats to public health and safety. Therefore, *F. tularensis* is classified as a Tier 1 select agent by the Federal Select Agent Program, which is managed jointly by the U.S. Department of Health and Human Services and U.S. Department of Agriculture [11]. The U.S. Environmental Protection Agency (USEPA) Homeland Security Research Program provides science and technology needed for effective response and recovery from natural, intentional, or accidental environmental catastrophes, including public health threats from microbial pathogens. During these events, numerous and diverse sample types (e.g. aerosol, surface, environmental) will need to be processed and analyzed, underscoring the need for sensitive, specific, quick, and straightforward methods to determine the extent of contamination and efficacy of remediation efforts. Although culturability is a traditional indicator of viability, *F. tularensis* isolation and identification by culture is challenging because the organism is highly infectious, fastidious, slow-growing (requiring incubations of up to 10 days), difficult to identify, and can be outcompeted in culture medium by other microorganisms present in environmental or clinical samples [10]. Thus, traditional culture methods for *F. tularensis* would not meet USEPA’s response and recovery needs during homeland security incidents.

Molecular detection methods such as polymerase chain reaction (PCR) cannot distinguish between culturable (potentially infectious) and inactivated pathogens since DNA from both types are present in the sample. However, features of PCR, such as rapidity, sensitivity, and specificity, can be combined with the requirement for growth in culture medium to quickly detect low concentrations of viable *Francisella* bacteria. Specifically, this culture-based PCR method is based on the change in PCR cycle threshold (ΔC_T_) which is calculated by subtracting the cycle threshold (C_T_) at time 0 (C_T0_, i.e. starting DNA levels) from the C_T_ value after incubation of samples in culture medium (C_Ti_, i.e. starting DNA levels in addition to those that have accumulated during the incubation): ΔC_T_ = C_T0_-C_Ti_ [12]. To help achieve the short incubation period required for early detection, an enhanced culture medium, composed of brain heart infusion broth supplemented with Vitox, Fildes, and histidine (BVFH), was previously developed for use in this method to promote early growth and enhanced cultivation of *F. tularensis* [13]. The method used in this study is similar to the rapid viability PCR method for detection of *F. tularensis* with the main differences being sample volumes, time points analyzed, and method of preparing the BVFH medium [14]. Other differences included the evaluation of (1) real environmental matrices (e.g. drinking water and well water), which are ecologically relevant to *F. tularensis*; (2) method sensitivity amidst high concentrations of inactivated target cells; and (3) more *F. tularensis* strains and PCR primer/probe sets to verify specificity and sensitivity for both Type A and B strains. These method parameters, not tested previously, would be relevant and important features during cleanup scenarios after an actual contamination event. Specifically, the bacterial Type would be unknown, high concentrations of inactivated and viable target organisms would be present, in addition to nontarget microorganisms and inhibitors found in the surrounding environment.

In this study, three Type A and B strains (Schu4, IN99, and Live Vaccine Strain [LVS]) were used to evaluate performance of this culture-based PCR method in various water matrices containing chemical and microbial challenges at low, mid, and high starting concentrations (1, 10, and 100 CFU mL^− 1^ targets, respectively). Chemical challenges included humic acid and metal oxides which could negatively affect *F. tularensis* growth, recovery from water samples, and/or interfere with downstream analyses. Various concentrations of inactivated *F. tularensis* cells were used as a microbial challenge to simulate post-decontamination/remediation scenarios and to determine the minimum concentrations and length of incubations required for viable cells to overcome the PCR signals from the inactivated or unculturable cells. Other microbial challenges included indigenous organisms present in well water, drinking water, and test dust to assess potential overgrowth and negative impact on the culture-based PCR detection of *F. tularensis*.

## Results

### Evaluation of PCR assays

Twelve strains of *Francisella* spp. representing Type A1 and A2, Type B, *F. novicida*, and *F. philomiragia* were used to determine specificity of three PCR assays targeting the conserved *Francisella fopA* gene, Type A-specific *pdpD* gene, and Type B-specific *ISFtu2* gene. Total genomic DNA was isolated from each of the twelve *Francisella* spp. strains and quantitative PCR using cellular standards were performed for each gene assay to confirm amplicon size, specificity, and sensitivity (Table 1). In agreement with previous studies, Type A strains Schu4, F2246, H3563, and KC1482 were positive for the *fopA* and *pdpD*, but not the *ISFtu2* PCR assays; while Type B strains LVS, IN99, KY99, OR96, and NY98 were positive for *fopA* and *ISFtu2* but not *pdpD* PCR assays [15–17]. Additionally, *F. novicida* and *F. philomiragia* were negative for all three PCR assays except for detection of the *fopA* gene in *F. novicida* as expected from a previous report [17]. For the Type A (Schu4) and Type B (IN99 and LVS) experimental samples, no statistical differences were observed between C_T_ values from the *fopA* and *pdpD* or *ISFtu2* gene assays (data not shown); thus, C_T_ values from the *fopA* gene assay were used and ΔC_T_ calculated as described in the Methods.

**Table 1:** *Francisella* spp. strains and specificity of PCR assays used in this study

***Francisella*** spp. strain	Description	Source	fopA (87 bp)	pdpD (104 bp)	ISFtu2 (118 bp)
*F. tularensis* subsp. *tularensis* Type A					
Schu4	Clinical isolate, Ohio, Type A1 strain	Laura Rose, CDC	+	+	–
F2246	Maryland isolate, Type A1 strain	Laura Rose, CDC	+	+	–
H3563	Oklahoma isolate, Type A1 strain	Laura Rose, CDC	+	+	–
KC1482	Utah isolate, Type A2, attenuated	Laura Rose, CDC	+	+	–
*F. tularensis* subsp. *holarctica* Type B					
LVS	Live vaccine strain, attenuated	Laura Rose, CDC	+	–	+
IN99	Indiana isolate	Laura Rose, CDC	+	–	+
NY98	Clinical isolate, New York	Laura Rose, CDC	+	–	+
OR96	Oregon isolate	Laura Rose, CDC	+	–	+
KY99	Kentucky isolate	Laura Rose, CDC	+	–	+
*F. novicida* strain U112^a^	Environmental isolate, saltwater	BEI Resources	+	–	–
*F. philomiragia* strain 25,015	Isolated from moribund muskrat, Utah	ATCC	–	–	–
*F. philomiragia* strain 25,018	Water isolate, Odgen Bay Refuge, Utah	ATCC	–	–	–

*CDC* Centers for Disease Control and Prevention, USA

*ATCC* American Type Culture Collection, USA

^a^The following isolate was obtained through the NIH Biodefense and Emerging Infections Research Resources Repository, NIAID, NIH: *Francisella novicida*, Strain Utah 112, NR-13

PCR amplicon/signal “+” detected or “-” not detected. Data are representative of three replicates

Sensitivity for each assay based on standard curves was 10 cell equivalents per mL

### Detection of *F. tularensis* in the presence of inactivated target cells

**Fig. 1:**
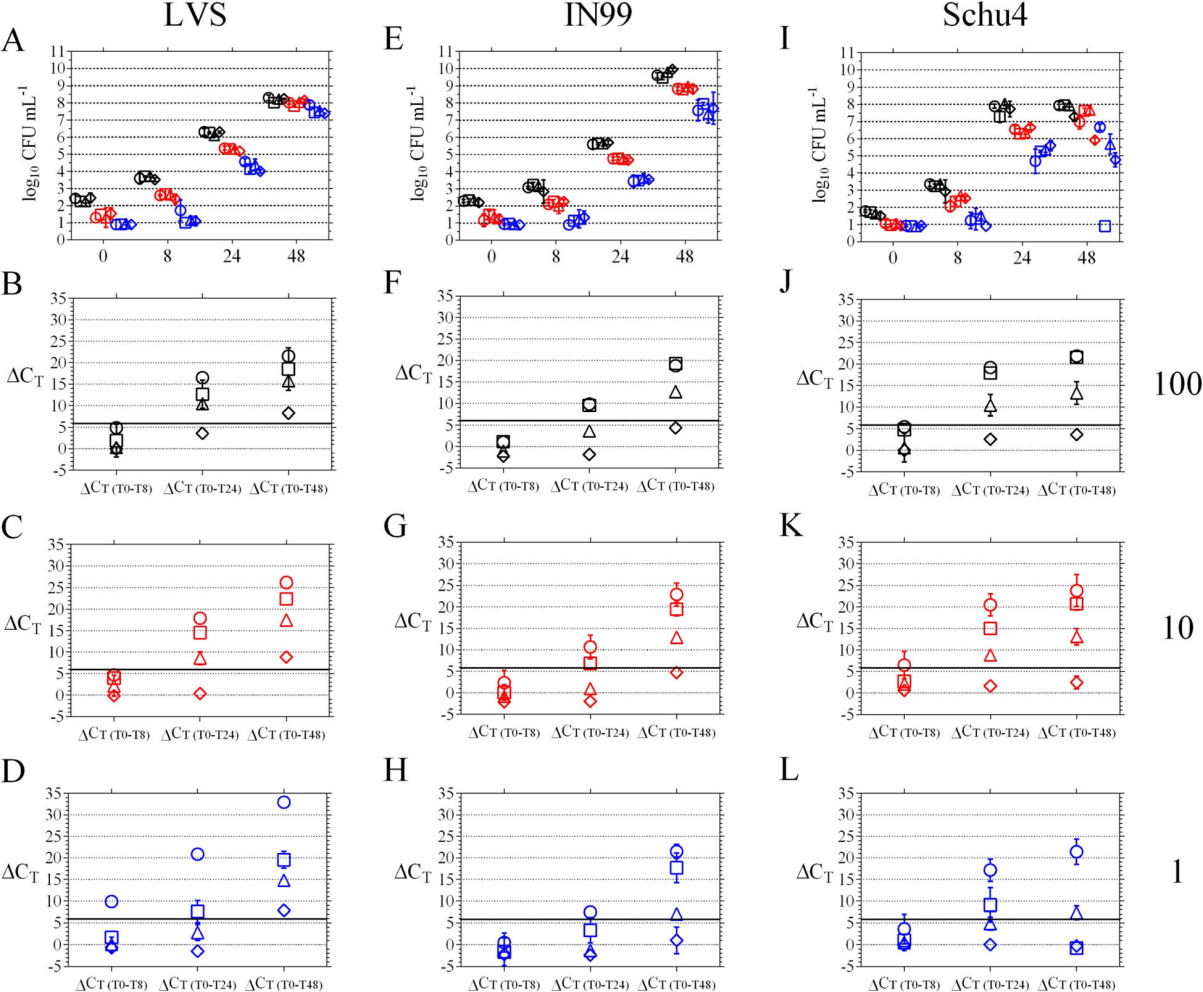
Detection of *F. tularensis* in the presence of inactivated target cells. *F. tularensis* strain LVS (**a-d**), IN99 (**e-h**), and Schu4 (**i-l**) were incubated in BVFH media containing 0 (circle), 10^2^ (square), 10^4^ (triangle), or 10^6^ (diamond) cells mL^− 1^ of their respective isopropanol-inactivated cells. For each inactivated target, culturable cells were added at targeted starting concentrations of 100 (black symbols), 10 (red symbols), and 1 (blue symbols) CFU mL^− 1^. Growth was monitored by CFU enumeration (**a**, **e**, and **i**) and results of the culture-based PCR assay are shown in the bottom three rows. ΔC_T_ of 6 is indicated by the bold black line

Inactivated target cells were generated by incubation in isopropanol for use in determining the minimum concentrations and length of incubations required for viable cells to overcome the PCR signals from their inactivated counterparts. Control wells containing 0, 10^2^, 10^4^, or 10^6^ of the inactivated IN99 and Schu4 cells, analyzed at 0, 8, 24, and 48 h, confirmed that these cells were not viable and that no change in PCR signal was observed over time (SFigure). In Fig. 1, data from the low, mid, and high targeted starting concentrations of 1, 10, and 100 colony forming units (CFU) mL^− 1^ are shown in blue, red, and black, respectively; and within each of those groups, open circles, squares, triangles, and diamonds represent samples that contained 0, 10^2^, 10^4^, and 10^6^ of inactivated cells, respectively. The actual starting concentrations (mean CFU mL^− 1^ ± standard deviation [SD]) for strain IN99 were 3 ± 1, 26 ± 9, and187 ± 77; and for strain Schu4 were ≤ 1, 8 ± 0, and 45 ± 7 for the low, mid, and high targets, respectively (Table 2). Actual starting concentrations for strain LVS was not performed. However, for each of the three targets, strains LVS, IN99, and Schu4 displayed expected CFU increases after 8, 24, and 48 h of incubation indicating growth was not inhibited in the presence of their respective inactivated cells (Fig. 1 a, e, and i). Because the actual titer of Schu4 was ≤1 for the low target, viable Schu4 cells may not have been inoculated into the wells of the 48-h plate containing 10^2^ inactivated Schu4 cells (Fig. 1 i, blue square, < limit of detection (LOD) of 1 log_10_ CFU mL^− 1^ for all three replicates).

**Table 2:** *F. tularensis* actual and targeted starting concentrations for each experiment

Experiment	Strain	Actual Starting Concentrations for each Targeted Concentration														
		Low					Mid					High				
		1 CFU mL^**− 1**^					10 CFU mL^**− 1**^					100 CFU mL^**− 1**^				
		Replicates			mean	SD	Replicates			mean	SD	Replicates			mean	SD
Inactivated target cells	LVS	–	–	–			–	–	–			–	–	–		
	IN99	2	4	2	**3**	1	20	36	22	**26**	9	193	260	107	**187**	77
	Schu4	< 1	< 1	1	**–**	–	8	8	8	**8**	0	48	51	37	**45**	7
Humic Acid	LVS	3	1	2	**2**	1	20	15	20	**18**	3	147	140	227	**171**	48
	IN99	< 1	< 1	< 1	**–**	–	2	2	< 1	**–**	–	17	9	7	**11**	5
	Schu4	2	2	1	**2**	1	27	31	20	**26**	6	TNTC	TNTC	TNTC	**–**	–
Arizona Test Dust	LVS	1	1	1	**1**	0	13	13	15	**14**	1	51	67	94	**71**	22
	IN99	–	–	–	**–**	–	–	–	–	**–**	–	–	–	–	**–**	–
	Schu4	–	–	–	**–**	–	–	–	–	**–**	–	–	–	–	**–**	–
Well Water	LVS	< 1	< 1	< 1	**–**	–	5	4	4	**4**	1	31	23	21	**25**	5
	IN99	1	1	2	**1**	1	10	15	12	**12**	3	61	73	83	**72**	11
	Schu4	1	1	1	**1**	0	12	11	13	**12**	1	123	107	103	**111**	11
Drinking Water	LVS	1	1	1	**1**	0	15	13	13	**14**	1	39	46	90	**58**	28
	IN99	4	4	4	**4**	0	33	42	46	**40**	7	287	400	300	**329**	62
	Schu4	< 1	1	< 1	**–**	–	9	8	7	**8**	1	41	47	41	**43**	3

“-”, not performed

*TNTC* too numerous to count

At the 100 CFU mL^− 1^ target, a ΔC_T_ ≥ 6 was achieved by 24 h in LVS and Schu4 samples containing ≤10^4^ inactivated cells (Fig. 1 b and j, respectively), except for IN99 where the mean ΔC_T(T0-T24)_ ± SD in the presence of 10^4^ inactivated cells was 3.6 ± 0.6 (Fig. 1 f, black open triangle). By 48 h, only strain LVS displayed the ΔC_T_ ≥ 6 for all samples (Fig. 1b), while strain IN99 and Schu4 had a mean ΔC_T(T0-T48)_ ± SD of 4.4 ± 1.2 and 3.7 ± 0.6 in the presence of 10^6^ inactivated target cells but ΔC_T_ ≥ 6 for all other samples (Fig. 1 f and j).

The same trend was observed at the 10 CFU mL^− 1^ target where the ΔC_T_ ≥ 6 was achieved by 24 h in the LVS and Schu4 samples (Fig. 1 c and k, respectively) containing ≤10^4^ inactivated cells and not for IN99, where the mean ΔC_T(T0-T24)_ ± SD in the presence of 10^4^ inactivated cells was 1.0 ± 0.3 (Fig. 1g, red open triangle). By 48 h, only strain LVS displayed the ΔC_T_ ≥ 6 for all samples (Fig. 1c), while strain IN99 and Schu4 had a mean ΔC_T(T0-T48)_ ± SD of 4.7 ± 0.3 and 2.4 ± 1.5 in the presence of 10^6^ inactivated target cells but ΔC_T_ ≥ 6 for all other samples (Fig. 1 g and k).

As expected, for the initial target concentration of 1 CFU mL^− 1^, a longer incubation period was needed to overcome the PCR signal detected from the inactivated target cells. A ΔC_T_ ≥ 6 at 24 h could not be achieved in samples containing > 10^2^ inactivated target cells for LVS and Schu4 (Fig. 1 d and l, respectively) while the ΔC_T_ ≥ 6 was only observed for IN99 samples in the absence of inactivated cells (Fig. 1h, blue open circle). After 48 h, ΔC_T_ ≥ 6 was achieved for all LVS samples (Fig. 1d) and for IN99 samples containing ≤10^4^ inactivated cells (Fig. 1h) in contrast to Schu4 where the ΔC_T_ ≥ 6 was only achieved in the presence of 10^4^, but not 10^2^, inactivated cells (Fig. 1l) most likely due to the low starting inoculum where certain samples may not have received any viable Schu4 cells (Table 2).

### *F. tularensis* detection in the presence of environmental inhibitors

#### Humic acid

Humic acid was used as a surrogate for natural organic matter to test for possible inhibition of *F. tularensis* growth and downstream PCR analyses. In these experiments, actual starting concentrations (mean CFU mL^− 1^ ± SD) for strain LVS were 2 ± 1, 18 ± 3, and 171 ± 48 CFU mL^− 1^ for the low, mid, and high targets, respectively (Table 2). For strain IN99, the starting inoculum was approximately 1 log_10_ unit lower for the low, mid, and high targets displaying ranges of < 1, < 1 to 2, and 7–17, respectively (Table 2). CFU could not be enumerated for Schu4 at the high target due to overgrowth; however, the mean CFU mL^− 1^ ± SD actual starting concentrations for the 1 and 10 CFU mL^− 1^ target were 2 ± 1 and 26 ± 6 CFU mL^− 1^, respectively (Table 2).

**Fig. 2:**
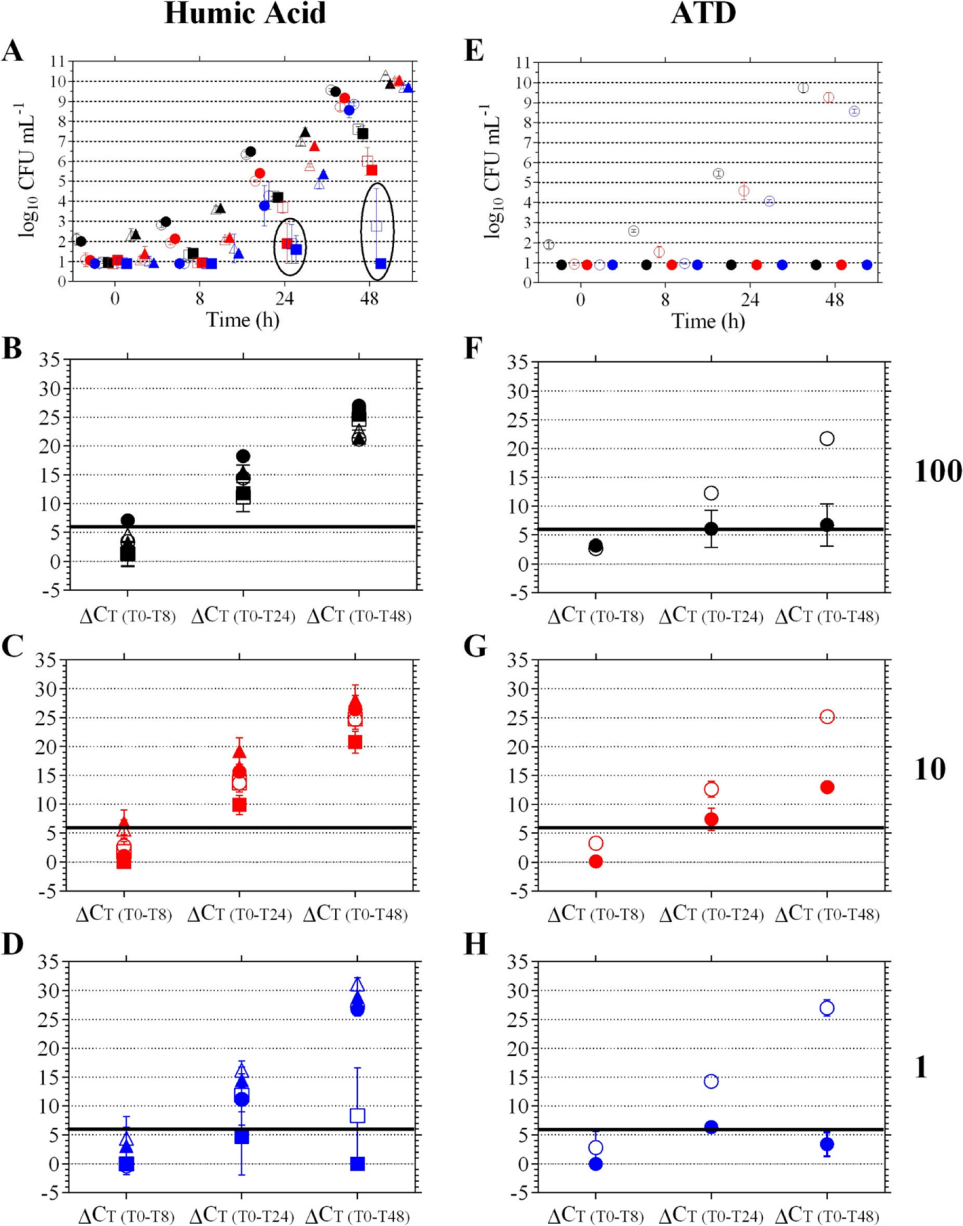
Detection of *F. tularensis* in the presence of humic acid and ATD. *F. tularensis* strain LVS (circles), IN99 (squares), and Schu4 (triangles) were incubated in BVFH containing either humic acid (**a-d**) or ATD (**e-h**) at targeted starting concentrations of 100 (black symbols), 10 (red symbols), and 1 (blue symbols) CFU mL^− 1^. Open symbols represent control wells with Milli-Q® water. Filled-in symbols represent wells with humic acid or ATD. Growth was monitored by CFU enumeration (**a** and **e**) and results of the culture-based PCR assay are shown in the bottom three rows. ΔC_T_ of 6 is indicated by the bold black line. Circled data points are referenced in the main text

Growth of *F. tularensis* strains LVS and Schu4 was not inhibited in the presence of 50 μg mL^− 1^ humic acid since CFU levels were not statistically different between their respective control and humic acid treated samples at each targeted starting concentration (*P* > 0.05, Fig. 2a, circle and triangle symbols). This observation was also true for strain IN99 at the high target (*P* > 0.05, Fig. 2a, open and filled black squares). Due to the low starting concentrations of IN99, it was possible that no cells were inoculated into some of the wells at the 1 and 10 CFU mL^− 1^ targets potentially giving false positives for humic acid inhibition of IN99 cell growth (Fig. 2a, circled data points). Furthermore, after 24 and 48 h of incubation for the 10 CFU mL^− 1^ target, the control IN99 samples had a mean log_10_ CFU mL^− 1^ ± SD concentration of 3.7 ± 0.2 and 6.0 ± 0.4, respectively (Fig. 2a, open red squares); while the 48-h incubated IN99 with humic acid samples had CFU levels of 5.6 ± 0.3 (Fig. 2a, filled red square) indicating growth of IN99 was not inhibited by humic acid.

Using the culture-based PCR method, a ΔC_T_ ≥ 6 was achieved following 24 h of incubation for LVS and Schu4 at each targeted starting concentration (Fig. 2 b-d, circle and triangle symbols, respectively) and for strain IN99 at the mid and high targets (Fig. 2 b and c, filled squares). Like the growth trends observed for strain IN99 at the 1 CFU mL^− 1^ target, the potential exclusion of viable IN99 cells into the samples may have resulted in an observed ΔC_T_ < 6, which could be a false positive indicator of humic acid inhibition (Fig. 2d, open and filled squares).

#### Arizona test dust

Arizona Test Dust (ATD) was used as an additional environmental test matrix to further evaluate F*. tularensis* detection with the culture-based PCR method. Strains IN99 and Schu4 were unable to be tested due to time and logistical constraints with the BSL-3 facility; thus, only the reference LVS strain was used for the ATD test matrix experiments. For LVS, actual CFU densities (mean CFU ± SD) were 1 ± 0, 14 ± 1, 71 ± 22, and for the low, mid, and high targeted starting concentrations, respectively, for each replicate (Table 2). For the samples containing ATD, LVS CFU levels were < limit of detection (LOD) because ATD background organisms overgrew the agar plate and *F. tularensis* colonies could not be identified (Fig. 2e, filled in symbols).

**Fig. 3:**
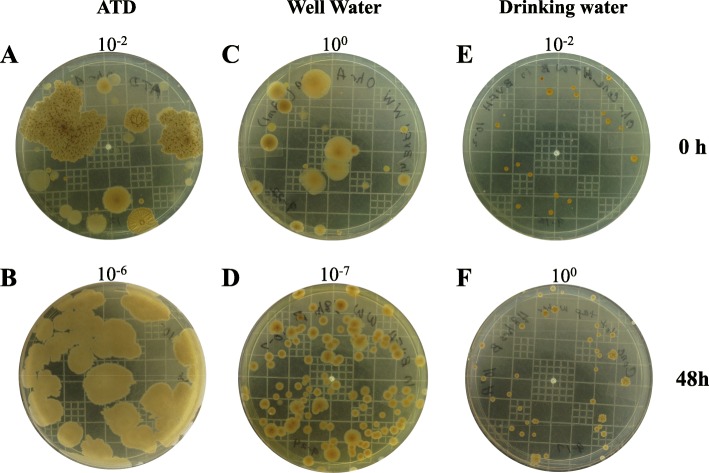
Heterotrophic growth from well and drinking water and ATD microorganisms in BVFH. Heterotrophic plate count analysis was performed on ATD (**a-b**), well water (**c-d**), and drinking water (**e-f**) control wells without *F. tularensis* after incubation in BVFH medium. Heart infusion agar plates shown here illustrate growth of background organisms after 0 (**a**, **c**, and **e**) and 48 h (**b**, **d**, and **f**) of incubation at the indicated dilutions. Images are representative of three replicates

Fig. 3 a and b show images of ATD microorganisms after incubation in BVFH media for 0 and 48 h, respectively. At 0 h, heterogeneous colony morphologies with various types of form, elevation, and margin was observed (Fig. 3a). In contrast, by 48 h, growth was dominated by a homogeneous mixture of microbes exhibiting large spreading colonies at high concentrations (Fig. 3b). ATD microbes exhibited rapid growth in the BVFH medium, with concentrations (mean log_10_ CFU mL^− 1^ ± SD) of 3.6 ± 0.0 at 0 h; 7.1 ± 0.6 at 24 h; and 7.8 ± 0.3 at 48 h. It is unclear from the culture data if ATD microbes inhibited LVS or masked their growth on agar plates. However, PCR results showed significantly lower levels of LVS in the presence of ATD compared to controls indicating that LVS growth was inhibited or outcompeted in the presence of ATD microbes (Fig. 2 f-h, *P* < 0.05, open versus filled in symbols at 24 and 48 h).

Although LVS growth was not detectable, the ΔC_T_ ≥ 6 was achieved in this experiment after 24 h for each targeted starting concentration (Fig. 2 f-h). Although the ΔC_T_ ≥ 6 was achieved by 24 h, the ΔC_T_ did not significantly increase by 48 h, for the mid and high targets, indicating growth of the LVS cells was compromised in the presence of ATD. At the 100 CFU mL^− 1^ target, ΔC_T_ ± SD values for the ATD containing samples at 24 and 48 h were 6.1 ± 3.2 and 6.8 ± 3.6, respectively (Fig. 2f, filled black circles). Similarly, at the 10 CFU mL^− 1^ target, ΔC_T_ ± SD values for the ATD containing samples at 24 and 48 h were 7.4 ± 1.9 and 13.0 ± 0.8, respectively (Fig. 2g, filled red circles). At the 1 CFU mL^− 1^ target, the ΔC_T_ ≥ 6 was achieved at 24 h but not at 48 h (mean ΔC_T_ ± SD of 6.3 ± 1.2 and 3.4 ± 2.1, respectively, Fig. 2h) indicating that inhibition or suppression of LVS growth by ATD microorganisms may be concentration dependent.

### Effect of indigenous microorganisms on *F. tularensis *detection in various water matrices

Since contaminated water sources have been implicated in tularemia outbreaks, well water and drinking water were used as additional environmental test matrices to evaluate the culture-based PCR method for *F. tularensis* detection. Microbial and chemical water quality data are presented for each well and drinking water experiment using strains LVS, IN99, and Schu4 (Table 3). Heterotrophic plate count (HPC) bacteria in well and drinking water ranged between 1.6–3.2 and < LOD to 3.2 log_10_ CFU mL^− 1^, respectively. Total coliforms and *Escherichia coli* levels in the well water ranged between 24 to 3650 and <  1 to 34 most probable number (MPN) per 100 mL, respectively (Table 3).

**Table 3:** Water quality measurements for well water and drinking water

Parameters^a^	Well Water experiments					Drinking water experiments				
	LVS	IN99	Schu4	mean	SD	LVS	IN99	Schu4	mean	SD
HPC (log_10_ CFU mL^−1^) on HIA	3.2	2.1	1.6	2.3	0.8	3.3	3.4	2.1	2.9	0.7
Total Coliforms (MPN 100 mL^− 1^)	3.6	1.6	1.4	2.2	1.2	–	–	–	–	–
*E. coli* (MPN 100 mL^− 1^)	34	< 1	< 1	–	–	–	–	–	–	–
pH^b^	7.39	7.27	7.57	7.41	0.15	8.39	8.17	8.54	8.37	0.19
temperature (°C)^b^	12.0	11.0	11.0	11.33	0.58	37.46	36.33	36.88	36.89	0.57
conductivity (μS cm^−1^)^b^	747	765	759	757	9	393	497	479	456	56
turbidity (NTU)	2.31	0.49	0.18	0.99	1.15	0.37	0.93	0.40	0.57	0.32
DO (%)	89.9	72.3	77.0	79.73	9.11	59.4	59.6	64.6	61.20	2.95
TOC	1.0	0.5	18.6	6.7	10.3	0.7	0.5	0.6	0.6	0.1
hardness^b^	460	410	462	444	29	171	188	170	176	10
alkalinity	324	259	–	–	–	87	93	93	91	4
Free Cl_2_	< 0.02	< 0.02	< 0.02	–	–	0.02	0.02	0.12	0.05	0.06
Total Cl_2_	0.02	0.02	0.02	0.02	0.00	0.08	0.07	0.24	0.13	0.10
NO_3_	0.82	0.93	1.00	0.92	0.09	0.74	0.47	0.51	0.57	0.15
PO_4_	0.15	0.05	0.67	0.29	0.33	0.24	0.29	0.34	0.29	0.05
Ca	281.8	115.8	114.7	170.8	96.2	30.7	36.4	36.5	34.5	3.3
Cu	< 0.001	< 0.001	< 0.001	–	–	0.05	0.08	0.04	0.06	0.02
Fe	0.09	0.03	0.02	0.05	0.04	0.07	0.14	0.05	0.09	0.05
K	2.2	1.8	1.8	1.9	0.2	2.3	3.0	2.9	2.8	0.4
Mg	13.8	15.3	14.6	14.6	0.7	9.3	13.2	12.6	11.7	2.1
Na	7.1	7.8	7.7	7.5	0.4	18.5	29.4	27.9	25.3	5.9
P	0.04	0.02	0.01	0.03	0.02	0.10	0.16	0.15	0.14	0.03
Si^b^	4.6	5.0	4.9	4.8	0.2	2.7	1.8	2.1	2.2	0.5
Zn	0.79	0.05	0.09	0.31	0.41	0.03	0.05	0.02	0.03	0.01

Abbreviations: *μS cm-1* microSiemens per centimeter, HIA heart infusion agar, *MPN* most probable number, *NTU* Nephelometric Turbidity Units, *SD* standard deviation

“-”, not performed

^a^units are mg L^−1^ unless stated otherwise

^b^*P* < 0.01 between well and drinking water samples. Unpaired t-tests were performed for each parameter except total coliforms, *E. coli*, free and total Cl_2_, and Cu

Among the chemical parameters measured, pH, temperature, conductivity, hardness, and silicon concentrations were statistically different between the two test water matrices (Table 3, *P* <  0.01) and most likely alkalinity as well, but this parameter was not measured for the Schu4 experiments, thus statistical analysis could not be performed. Well and drinking water HPC bacteria displayed heterogeneous colony morphologies at 0 h (Fig. 3 c and e, respectively); however, by 48 h, HPC growth was comprised of a less diverse subset of microbes with well water HPC bacteria growing to high concentrations (Fig. 3d, 10^− 7^ dilution of well water versus Fig. 3f, 10^0^ dilution of drinking water). Similar to the ATD microbes, those in well water exhibited rapid growth in the BVFH medium reaching high CFU concentrations by 48 h. Specifically, well water HPC levels (mean log_10_ CFU mL^− 1^ ± SD) after 0, 24, and 48 h of growth were 1.7 ± 0.3, 8.2 ± 0.2, and 9.0 ± 0.2, respectively, compared to drinking water HPC levels of 3.3 ± 0.4, 1.8 ± 0.2, and 2.8 ± 0.3 at the same time points.

### Well water

**Fig. 4:**
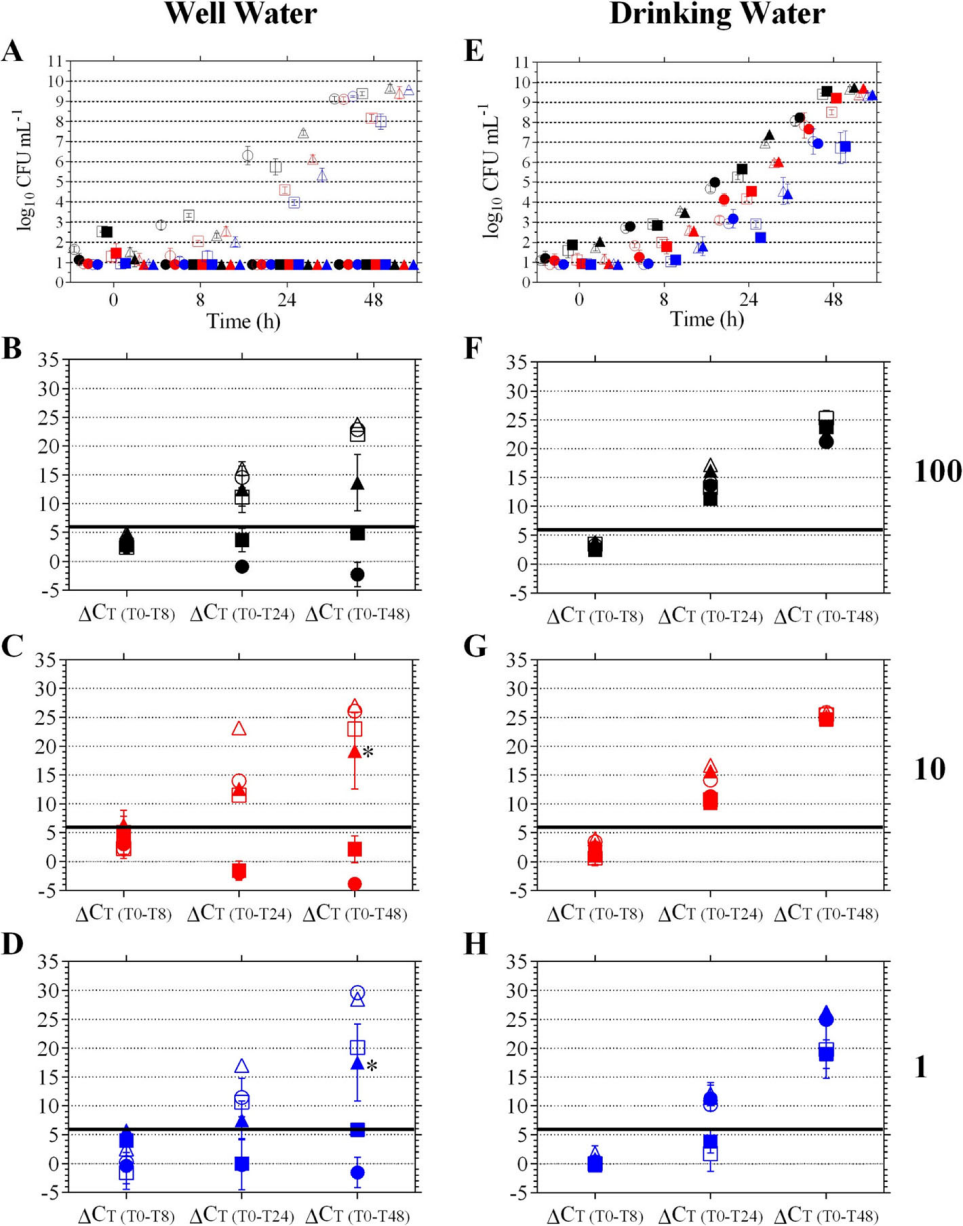
Detection of *F. tularensis* in well and drinking water matrices. *F. tularensis* strain LVS (circles), IN99 (squares), and Schu4 (triangles) were incubated in BVFH containing either well water (**a-d**) or drinking water (**e-h**) at targeted starting concentrations of 100 (black symbols), 10 (red symbols), and 1 (blue symbols) CFU mL^− 1^. Open symbols represent control wells with sterilized well or drinking water. Filled symbols represent wells with well or drinking water. Growth was monitored by CFU enumeration (**a** and **e**) and PCR results are shown in **b-d** and **f-h**. ΔC_T_ of 6 is indicated by the bold black line. *, *P* < 0.05 Schu4 v IN99 well water samples

Actual starting densities (mean CFU ± SD) for strain LVS were <  1, 4 ± 1, and 25 ± 5; for IN99 were 1 ± 1, 12 ± 3, and 72 ± 11; and for Schu4 were 1 ± 0, 12 ± 1, and 111 ± 11 for the low, mid, and high targets, respectively (Table 2). For samples containing well water, CFU levels for all three *F. tularensis* strains were < LOD because well water microbes overgrew the agar plate and *F. tularensis* colonies could not be identified (Fig. 4a, filled in symbols). In contrast, control samples containing sterilized well water (sWW), growth of strains IN99 and Schu4 was observed at 8, 24, and 48 h in a starting concentration dependent manner as expected (Fig. 4, open squares for IN99 and triangles for Schu4, with the low, mid, and high targets shown in blue, red, and black, respectively). Except for the 24 h time point at the 1 and 10 CFU mL^− 1^ target, LVS cells displayed the same growth pattern throughout the time course (Fig. 4a, missing red and blue open circles at 24 h, but present at all other time points). Those 24 h CFU values could not be determined due to the dehydration of agar plates for a subset of the serially diluted samples while the remaining subset were contaminated with morphologically different colonies than those observed with the LVS in well water samples.

For control, sWW samples, a ΔC_T_ ≥ 6 was observed at 24 and 48 h for each strain at all targeted starting concentrations (Fig. 4 b-d open symbols). However, for strains LVS and IN99 in the well water samples, the mean ΔC_T_ was < 6 and ranged from − 3.8 to 3.1 for LVS and − 1.6 to 5.8 for IN99 for all targeted concentrations and time points (Fig. 4 b-d, filled circles and squares). Notably, for strain Schu4, a ΔC_T_ > 6 was observed at 24 h for the high (mean ΔC_T_ ± SD, 12.5 ± 4.0) and mid (12.6 ± 0.7) targets and by 48 h for the low target (17.5 ± 6.7) (Fig. 4 b-d, filled triangles). Moreover, although actual starting concentrations between strain IN99 and Schu4 were not statistically different at the low and mid targets (*P* > 0.3, Table 2), ΔC_T(T0-T48)_ values were statistically different between the two strains (Fig. 4 c-d, * *P* <  0.05) collectively indicating that early detection of *F. tularensis* in well water type matrices may be strain dependent.

### Drinking water

In contrast to well water, drinking water microorganisms did not significantly amplify in the BVFH medium (mean log_10_ CFU mL^− 1^ ± SD: 3.3 ± 0.4 at 0 h vs 2.8 ± 0.3at 48 h, *P* > 0.05 and Fig. 3d vs 3f). In the absence of fast growing HPC organisms, growth of strains LVS, IN99, and Schu4 was observed in the presence of drinking water in a starting concentration dependent manner as expected (Fig. 4e). Moreover, there were no statistical differences between CFU levels in the sterilized drinking water (sDW) controls and the levels of each strain in their respective drinking water samples at each targeted starting concentration and time point (*P* > 0.05, Fig. 4e). Actual starting densities (mean CFU ± SD) for strain LVS were 1 ± 0, 14 ± 1, and 58 ± 28; for IN99 were 4 ± 0, 40 ± 7, and 329 ± 62; and for Schu4 were <  1 to 1, 8 ± 1, and 43 ± 3 for the low, mid, and high targeted concentrations, respectively (Table 2).

The mean ΔC_T(T0-T24)_ ± SD at the 100, 10, and 1 CFU mL^− 1^ targeted dose was, respectively, 13.6 ± 0.5, 11.3 ± 0.4, and 11.3 ± 1.1 for strain LVS; 16.2 ± 0.4, 15.7 ± In the absence of fast growing HPC0.9, and 11.4 ± 2.2 for strain Schu4; and 11.3 ± 0.6, 10.2 ± 0.3, and 3.8 ± 3.3 for strain IN99 (Fig. 4 f-h). Thus, the ΔC_T_ ≥ 6 was achieved following 24 h of incubation in the presence of drinking water for each strain at each targeted dose, except for strain IN99 at the 1 CFU mL^− 1^ target (Fig. 4h, open and filled blue squares, control and drinking water samples, respectively). Because the sDW controls were also below the ΔC_T_ of 6 and lower IN99 CFU levels were observed at the 24 h time point compared to LVS and Schu4 (Fig. 4e, blue squares vs blue circles and triangles, respectively), the ΔC_T_ < 6 was most likely not due to inhibition of IN99 growth in the drinking water matrix. By 48 h, a ΔC_T(T0-T48)_ ± SD of 19.0 ± 4.3 at the low target was observed for strain IN99 (Fig. 4h, filled blue square).

## Discussion

In this study, the criteria used for early detection of *F. tularensis* growth is based on the change in PCR cycle threshold (ΔC_T_) which is calculated by subtracting the cycle threshold (C_T_) at time 0 (C_T0_, i.e. starting DNA levels) from the C_T_ value after incubation of samples in culture medium (C_Ti_, i.e. starting DNA levels in addition to those resulting from growth of viable cells): ΔC_T_ = C_T0_-C_Ti_. For detection of *F. tularensis* cells, a ∆C_T_ value ≥6 was chosen to indicate growth of viable cells during the incubation period and was similarly used as a criterion for early detection of *Bacillus anthracis* and *Yersinia pestis* growth [12, 18]. It should be noted that this ΔC_T_ nomenclature is different from the one commonly used in gene expression studies employing the comparative C_T_, or 2^- ΔΔ*CT*^, method where ΔC_T_ is the difference in threshold cycle between target and reference genes within the same sample [19].

Various *F. tularensis* PCR assays were tested for use in this method. The *fopA* gene encodes an outer membrane associated protein, *pdpD* encodes the pathogenicity determinant protein D, and *ISFtu2* encodes a multiple copy, insertion element-like sequence [15, 16]. The *pdpD* gene lies within a putative operon consisting of *pdpDiglABCD* and encodes a 140.7-kDa membrane-associated protein that is required for virulence but not intracellular growth [20, 21]. However, in *F. tularensis* subsp. *holarctica* (Type B) strains, most of the *pdpD* gene is missing or absent and may play a role in the difference in virulence between Type A and B strains [22]. The assay for the *pdpD* gene is absent from Type B strains and contains a 144 bp insert in *F. novicida* strains [15] which was verified in this study (Table 1). No statistical differences were observed for C_T_ values collected from the *fopA* and *pdpD* assays for Schu4 and between the *fopA* and *ISFtu2* assays for IN99 and LVS samples (*P* > 0.05, data not shown); thus, for consistency, C_T_ values from the *fopA* PCR assay were reported and analyzed here for strains Schu4, IN99, and LVS.

To evaluate performance under conditions mimicking post-decontamination scenarios, the culture-based PCR method was tested in the presence of low titers of viable target cells and high levels of inactivated target cells which may be present after remediation efforts. Although different results were obtained for each strain, the overall trend indicated the assay can detect growth of 1 viable cell mL^− 1^ by 48 h in the presence of up to 10^4^ inactivated cells mL^− 1^ for strain IN99 and Schu4 (Fig. 1 h and l, respectively) and up to 10^6^ inactivated cells mL^− 1^ for strain LVS (Fig. 1d). Moreover, at low inactivated background levels of 10^2^–10^4^ cells mL^− 1^, a ΔC_T_ ≥ 6 was achieved for all strains after 24–48 h incubation in BVFH. However, for low titers of live target cells in the presence of high levels of inactivated target cells, an incubation period > 48 h may be needed to observe a ΔC_T_ ≥ 6 yet would still be less time required for identification using traditional culture methods.

Humic acids are reported to bind DNA in a sequence-specific manner, thus limiting the amount of template DNA available for the PCR reaction [23]. Due to the difficulty in measuring humic acid concentrations directly in water matrices, their concentrations are usually estimated from total organic carbon (TOC) or dissolved organic carbon (DOC) measurements. An Indiana-based study monitored DOC concentrations over a 22-month period in river water before and after conventional drinking water treatment and found DOC concentrations to range between 2 and 12 μg mL^− 1^ in river water and 1–4 μg mL^− 1^ in chloraminated, finished water [24]. Thus, even if humic acids constituted a majority of the DOC, the humic acid concentration evaluated in this study, 50 μg mL^− 1^, was between 4 and 50 times higher than levels reported for surface and finished drinking water samples. Thus, the evaluation of this high concentration of humic acid in this study represented a worst-case scenario of the possible concentration and carry-over of environmental inhibitors into samples for processing and downstream molecular analyses. Without prior removal of the humic acid from DNA extracted samples, PCR reactions were inhibited (data not shown). Thus, these samples required a clean-up step after DNA isolation, after which PCR results were not statistically different from controls with no humic acid (P > 0.05, Fig. 2 b-d). For this chemical challenge, results from the method indicated the ΔC_T_ ≥ 6 was achieved following 24 h of incubation for LVS and Schu4 at each targeted starting concentration and for strain IN99 at the mid and high targets (Fig. 2 b-d). Collectively, despite the issues with the IN99 inoculum, the method was able to detect early amplification of *F. tularensis* in the presence of humic acid after 24 h of incubation.

Arizona test dust (ATD) is commonly used as a surrogate for atmospheric particles, soil, and household dust due to its consistent particle size distribution and chemical and biological composition [25–27]. Thus, ATD was used as a test matrix for its representation of environmental challenges possibly encountered during environmental sampling and effects on the culture-based PCR method. In the 4 mg mL^− 1^ ATD concentration used in this study and based on the lowest composition percentages of the metal oxides or ions (i.e. 10% Al_2_O_3_, 2% Fe_2_O_3_, and 0.51% TiO_2_), the ATD samples contained 210 μg mL^− 1^ of Al, 56 μg mL^− 1^ of Fe, and 20 μg mL^− 1^ of TiO_2_, which is approximately 25–400 times above the PCR inhibitory levels previously reported for each inhibitor [28, 29]. Moreover, silicon, the main constituent of ATD (68–76% SiO_2_) was previously reported to inhibit PCR by interfering with Taq polymerase [30]. Thus, similar to humic acid, ATD samples required a clean-up step for extracted DNA prior to PCR analysis in order to remove the multiple PCR inhibitors contained within ATD (data not shown).

In addition to chemical challenges, previous microbial analysis of ATD indicated a composition of yeast, molds, actinomycetes, *Bacillus* spp., *Micrococcus* spp., and *Streptomyces* spp. with HPC concentrations of 4.6 log_10_ CFU 10 mg^− 1^ on Reasoner’s 2A agar (R2A) [31], which was approximately 1 log_10_ higher than levels observed in this study (3.6 ± 0.06 log_10_ CFU 10 mg^− 1^ on R2A, Methods Section). In this study, CFU enumeration of LVS was not possible in the presence of fast-growing, competing ATD microbes (Fig. 2e, filled circles v control, open circles). Moreover, although a mean ΔC_T_ ≥ 6 was observed for LVS at each targeted starting dose, method performance was concentration dependent since mean ΔC_T_ ± SD values at 24 h decreased by 48 h at the low target (Fig. 2h, filled circles 6.3 ± 1.2 to 3.4 ± 3.1, respectively) and only increased slightly from 24 to 48 h at the high target (Fig. 2f, filled circles, 6.1 ± 3.2 to 6.8 ± 3.6, respectively). Nonetheless, the culture-based PCR method was able to detect LVS growth after 24 h of incubation demonstrating the specificity and sensitivity of PCR analysis amidst a high interfering background and negative results from traditional culture methods.

Similarly, for the well water test matrix, *F. tularensis* CFU densities could not be quantified at 8, 24, and 48 h post incubation due to the rapid growth of well water microbes (Fig. 4a). Acid pretreatment of environmental samples has been shown to reduce interference by indigenous microbes and enhance recovery of *F. tularensis* from natural water samples [32, 33]. However, no significant differences in HPC densities were observed between acid treated well water samples and non-treated controls (mean log_10_ CFU mL^− 1^ ± SD: 0.642 ± 0.11 and 0.635 ± 0.37, respectively on heart infusion agar (HIA); *P* = 0.6; four replicates each). Although the fast-growing, competing microbes negatively affected growth and enumeration of *F. tularensis* in the well water samples, the culture-based PCR method detected early growth of the Type A1 Schu4 strain, in contrast to the Type B strains LVS and IN99 (Fig. 4 b-d).

Although the persistence and growth in natural waters for both types have been previously reported [reviewed in [6]], it is unknown whether environmental growth fitness and survival mechanisms differ between Type A and B strains. Infections with Type A1 strains result in higher mortality rates compared to Type A2 and Type B strains [9, 34]. Thus, virulence mechanisms present in Schu4, but absent in LVS and IN99, could confer a growth/survival advantage as potentially observed in the well water experiments where only Schu4 growth was detected amongst a high background of competing microbes (Fig. 4 b-d). For future culture-based PCR method studies, more avirulent and virulent *F. tularensis* Type A and B strains should be further tested in the well water and ATD matrices to confirm this interesting observation of potentially strain dependent fitness to compete with fast-growing microbes.

The occurrence of coliform bacteria in chlorinated systems was reported to be significantly higher at temperatures above 15 °C than at 0–15 °C [35]. Thus, to assess the performance of the culture-based PCR assay for *F. tularensis* in the presence of drinking water microorganisms, warm tap water (36.9 ± 0.6 °C, Table 3) was concentrated 100-fold to collect a high titer of indigenous drinking water microbes. Their presence in the BVFH medium neither inhibited nor outcompeted *F. tularensis* growth (Fig. 4e) and did not negatively affect the early detection of *F. tularensis* using the culture-based PCR assay (Fig. 4 f-h).

## Conclusions

In this study, a culture-based PCR method was able to detect early growth of low levels of *F. tularensis* cells amongst high levels of their respective inactivated target cells and various environmental interferences and inhibitors. Results also indicated that indigenous drinking water organisms were not capable of growth in nutrient rich medium (e.g. BVFH), unlike those in ATD and well water. Thus, this method could also be used to detect early growth of other water-based pathogens, such as *Legionella pneumophila*, where drinking water is an important source of human exposure [36]. The culture-based PCR method evaluated in this study can be used as an alternative to traditional culture methods for detection and identification of fastidious pathogens by combining the rapidity, sensitivity, and specificity of PCR with culturability as an indicator of microbial viability.

## Methods

### Bacterial strains and growth conditions

Table 1 lists the bacterial strains used in this study. Stock and stationary phase cultures of *Francisella* spp. were prepared as previously described by Morris et al. [13]. For the test conditions described below, *F. tularensis* strains LVS, IN99, and Schu4 were grown in a previously developed enhanced growth medium, BVFH, for *Francisella* spp., which consisted of brain heart infusion broth (Becton Dickinson [BD] Biosciences, USA) supplemented with 2% Vitox™ (Oxoid Hampshire UK), 10% Fildes (Remel, USA), and 1% _L_-histidine (Fisher Scientific, USA) [13]. Experiments with virulent *F. tularensis* strains were conducted under biosafety level 3 (BSL-3) conditions at the University of Cincinnati College of Medicine with protocols approved by the university’s Institutional Biosafety Committee and the Select Agent Program.

### Colony forming units (CFU) enumeration

To determine *Francisella* spp. densities, an aliquot of the bacterial suspension was serially diluted in Butterfield’s buffer (BB; 42.5 mg monopotassium phosphate L^− 1^; Hardy Diagnostics, USA) and plated on chocolate agar plates (BD Biosciences, USA). Plates were incubated for 48 h [for spot plate method [37, 38]] or 4–7 d [for spread plate method [39]] at 37 °C. The spot plate method was used for evaluation of high CFU titers at later time points. The limit of detection (LOD) was 1 and 1.95 log_10_ CFU mL^− 1^, for the spread and spot plate method, respectively.

Heterotrophic plate count (HPC) bacteria were enumerated by the spread plate method on heart infusion agar (HIA, Difco Laboratories) following incubation at 35 °C for 7 d or Reasoner’s 2A agar (R2A, Difco Laboratories) following incubation at 28 °C for 7 d. The LOD was 1 log_10_ CFU mL^− 1^. For untreated well water samples only, total coliforms and *Escherichia coli* were measured using Colilert® (Idexx Laboratories, USA) following manufacturer’s protocols.

### Preparation of *F. tularensis* cell suspensions

Bacterial cultures were grown in triplicate for each strain to stationary phase, washed by centrifugation (3000 relative centrifugal force [RCF] for 10 min at 4 °C), and stored in BB for 24 h at 4 °C. These resting cells were used as the inoculum to more closely mimic the physiological state of planktonic cells in the natural aquatic environment [40]. Suspensions were enumerated for CFU and the concentrations before and after incubation at 4 °C were not statistically different (*P* > 0.05, STable). Resting cells cultures were diluted in BVFH to yield approximate suspensions of 15, 150, and 1500 CFU mL^− 1^ and for use in experiments, these suspensions were diluted 15-fold in the various matrices to achieve targeted starting concentrations of 1, 10, and 100 CFU mL^− 1^, respectively. Actual starting concentrations are listed in Table 2 and were enumerated by spread plating 200 μL onto five separate chocolate agar plates (BD Biosciences, USA) resulting in a LOD of 1 CFU mL^− 1^.

### Collection of water samples

Potable municipal drinking water samples were collected from laboratory taps and were derived from river water treated by coagulation/flocculation/sedimentation, rapid sand filtration, granular activated carbon filtration, and chlorination. Ten liters were concentrated by filtration through a 0.45 μm polycarbonate filter (GE Osmonics, USA) and resulted in a 100 mL volume of 100-fold concentrated drinking water. A 1 mL aliquot of a 10% (w/v) sodium thiosulfate (Sigma-Aldrich, USA), prepared using distilled water, was added to the concentrate to neutralize disinfectant residuals.

Well water samples were collected on a private farm from a well supplying nonpotable ground water from a deep aquifer. The well water pump was operated for a few minutes before two 1 L samples were collected in sterile collection bottles. Water temperature was recorded right after sampling with a sterilized thermometer. Samples were kept at 4 °C for up to 4 h before transport to the laboratory. A 10 mL portion of the drinking and well water was filtered through a 0.1 μm Supor® (hydrophilic polyethersulfone) membrane Acrodisc® syringe filter (Pall Corporation, USA) resulting in sterilized drinking water (sDW) and well water (sWW) for use as controls.

### Preparation of humic acid and Arizona test dust (ATD)

A 750 μg mL^− 1^ solution of humic acid was prepared using Milli-Q® water (EMD Millipore, USA) and was added to samples at a final concentration of 50 μg mL^− 1^. Arizona test dust (ATD, Powder Technologies Inc., USA) was chemically composed of 68–76% SiO_2_, 10–15% Al_2_O_3_, 2–5% CaO, 2–5% Fe_2_O_3_, 2–5% K_2_O, 2–4% Na_2_O, 1–2% MgO, and 0.5–1% TiO_2_. Previously published analysis of ATD indicated a microbial composition consisting of yeast, molds, actinomycetes, *Bacillus* spp., *Micrococcus* spp., and *Streptomyces* spp. and HPC concentrations of 3.9, 4.6 and 4.7 log_10_ CFU per 10 mg on Sabouraud dextrose agar, R2A, and Trypticase soy agar plus 5% sheep blood, respectively [31]. In this study, heterotrophic plate count (HPC) bacteria in ATD was 3.9 ± 0.05 and 3.6 ± 0.06 log_10_ CFU 10 mg^− 1^ on HIA and R2A, respectively. A 60 mg mL^− 1^ solution of ATD was prepared using Milli-Q® water and was added to samples at a final concentration of 4 mg mL^− 1^.

### Preparation of inactivated *F. tularensis* cell suspensions

For each *F. tularensis* strain, static cultures were pelleted and washed twice by centrifugation at 3000 rcf for 10 min at 21 °C with 25 mL of BB. Aliquots of the suspensions were incubated in either BB (for controls) or 70% isopropanol (for inactivation) at 21 °C for 2 h. Suspensions were then washed twice by centrifugation and resuspended with 20 mL of BB. Three control and isopropanol treated replicates were performed for each strain. To confirm inactivation, suspensions were enumerated for CFU by spread plating 200 μL onto five separate chocolate agar plates (BD Biosciences, USA) resulting in a LOD of 1 CFU mL^− 1^. For control suspensions, the mean log_10_ CFU mL^− 1^ ± SD concentrations for LVS, IN99, and Schu4 were 6.9 ± 0.1, 7.0 ± 0.0, and 6.7 ± 0.1, respectively. CFU concentrations for all inactivated suspensions were < LOD. For use in experiments, the inactivated cell suspensions were diluted (based on the concentrations of the respective strain’s control suspensions) and added to samples to yield a final concentration of 10^2^, 10^4^ or 10^6^ inactivated cells mL^− 1^.

### Water quality analysis

Drinking and well water samples were analyzed for free and total chlorine (Cl_2_), pH, temperature, conductivity, percent dissolved oxygen (DO), hardness, and turbidity (Table 3) [41]. Measurements were made using DPD colorimetric analysis for free and total chlorine; a glass electrode 440 pH meter (Corning® Electrochemistry Products) for pH; a YSI 566 Multi Probe System (YSI Environmental) for temperature, conductivity, and DO; ethylenediaminetetraacetic acid titration for hardness (Hach Company); and a portable 2100 Turbidimeter (Hach, USA) for turbidity. Additionally, water samples were submitted to the National Risk Management Research Laboratory at the US EPA in Cincinnati, OH to assay total organic carbon (TOC) (EPA Method 415.3 rev 1.1); trace metals (EPA Method 200.7); phosphate (PO_4_) (EPA Method 365.1); and nitrate (NO_3_) (EPA Method 353.2) (Table 3).

### Isolation and preparation of total DNA

DNA was extracted from bacterial cells using the MasterPure™ Complete DNA purification kit (Epicentre Biotechnologies Inc., USA) according to manufacturer’s protocol. The DNA pellet was resuspended in 50 μL of molecular grade water. To remove PCR inhibitors from samples containing humic acid and ATD, an additional purification step was performed by using the OneStep™ PCR Inhibitor Removal Kit (Zymo Research, USA) following manufacturer’s protocols. Final DNA concentrations were measured with a Nanodrop ND-1000 Spectrophotometer (Thermo Scientific, USA) and analyzed as described below.

### Incubation of *F. tularensis* in the presence of each test matrix

Ninety-six deep well polypropylene plates (Fisher Scientific, USA), with a working volume of 1.8 mL per well, were pre-loaded with a sterile 4 mm glass bead per well (to aid in aeration of the samples). In each well, 0.1 mL of the *F. tularensis* suspensions and 0.1 mL of suspensions containing either inactivated target cells, drinking water, well water, humic acid, or ATD (except for control wells), was added to 1.3 mL of BVFH in triplicate. For control wells, 0.1 mL of BB, sDW, sWW, or Milli-Q®, for the humic acid and ATD experiments, was used. Duplicate wells without bacteria (0.1 mL of BB) for each test matrix were also included. The final volume in each well was 1.5 mL where both bacterial cells and test matrix or controls were diluted 15-fold.

For each experiment, four 96-well plates were generated for each time point (0, 8, 24, and 48 h) to minimize the loss of volume due to evaporation and sampling for culture and molecular analyses. After samples were added, plates were covered with an air-permeable AeraSeal™ (Excel Scientific Inc., USA) and incubated at 37 °C with shaking (Innova® 42 shaker/incubator, New Brunswick Scientific, USA). After each time point, 1 mL from each well was transferred to 1.7 mL tubes, processed for DNA isolation, and analyzed by real-time PCR. The remaining volume (approximately 400 μL) in the well was used for CFU enumeration.

### PCR assay

Forward (F) and reverse (R) primers and probe (Pr) used for *Francisella* detection were F: 5-AAC AAT GGC ACC TAG TAA TAT TTC TGG-3′, R: 5′-CCA CCA AAG AAC CAT GTT AAA CC-3′, and Pr: 5’FAM-TGG CAG AGC GGG TAC TAA CAT GAT TGG T-BHQ1 3′ for fopA, an 87 base pair (bp) amplicon [16]; F: 5′- GAG ACA TCA ATT AAA AGA AGC AAT ACC TT-3′, R: 5′- CCA AGA GTA CTA TTT CCG GTT GGT-3′, Pr: 5’FAM-AAA ATT CTG CTC AGC AGG ATT TTG ATT TGG TT-BHQ1 3’for pdpD, a 104 bp amplicon [15]; and F: 5′- CTT GTA CTT TTA TTT GGC TAC TGA GAA ACT-3′, R: 5′- CTT GCT TGG TTT GTA AAT ATA GTG GAA-3′, Pr: 5’FAM-AC CTA GTT CAA CCT CAA GAC TTT TAG TAA TGG GAA TGT CA-BHQ1 3’for ISFtu2, a 118 bp amplicon [15]. A total reaction volume of 20 μL contained 10 μL 2x TaqMan Universal Master Mix (Applied Biosystems, USA), 200 nM primer concentrations, 6.4 nM probe concentration, and 2 μL total DNA. The thermal cycling conditions, performed on the QuantStudio™ 6 Flex Real-Time PCR System (ThermoFisher Scientific, USA) included a pre-denaturation step at 50 °C for 2 min and 95 °C for 10 min; 45 cycles of denaturation at 95 °C for 10 s and annealing and extension at 60 °C for 30 s; and a final hold at 45 °C for 5 min. For initial confirmatory tests, 10-fold serial dilutions of *F. tularensis* KC1482 (Type A2 strain) and LVS (Type B strain) DNA ranging from 1 to 10^7^ cell equivalents mL^− 1^ were used in the PCR assays and analyzed in triplicate to determine sensitivity of each gene assay.

The change in PCR cycle threshold (ΔC_T_) which is calculated by subtracting the cycle threshold (C_T_) at time 0 (C_T0_) from the C_T_ value after incubation of samples in the culture medium (C_Ti_): ΔC_T_ = C_T0_-C_Ti_ [12]. This was annotated on the x-axis as ΔC_T (T0-T8)_, ΔC_T (T0-T24)_, and ΔC_T (T0-T48)_. For the T_0_ cases where no PCR response was obtained (undetermined results), the C_T_ values were set to 45 (since 45 PCR cycles were used). For detection of viable *F. tularensis* cells, a ∆C_T_ value ≥6 was chosen to indicate an increase in DNA concentration resulting from growth during the incubation period.

### Statistical analysis

Statistical significance was determined with an unpaired, t test with corrections for multiple comparisons made using the Holm-Šídák method. *P* values < 0.05 were considered statistically significant. For figures, data are expressed as mean with standard deviation. Statistical analyses and generation of graphs were performed using Prism 6 (GraphPad Software, USA).

## Supplementary information

**Additional file 1: SFigure.** Control wells containing no live *F. tularensis* in the presence of inactivated target cells. Wells containing no live IN99 (A) or Schu4 (B) cells but 0 (circle), 10^2^ (square), 10^4^ (triangle), or 10^6^ (diamond) of the respective isopropanol inactivated target cells confirmed that treated cells were not viable and that no change in PCR signal was observed over time. Data are from three replicates and presented as mean ΔC_T_ values with error bars indicating standard deviation.

**Additional file 2: STable.***F. tularensis* resting cell concentrations

## Data Availability

Data generated for this study is publicly available as per U.S. EPA policy.

## Abbreviations

ATCC: American Type Culture Collection
ATD: Arizona Test Dust
BB: Butterfield’s buffer
BEI: Biodefense and Emerging Infections Research Resources Repository
BVFH: Brain heart infusion broth supplemented with Vitox, Fildes, and histidine
CDC: Centers for Disease Control and Prevention
CFU: Colony forming units
C_T_: Cycle threshold
ΔC_T_: Change in PCR cycle threshold
DO: Dissolved oxygen
DOC: Dissolved organic carbon
F: Forward
HIA: Heart infusion agar
HPC: Heterotrophic plate count
LOD: Limit of detection
LVS: Live Vaccine Strain
MPN: Most probable number
NIAID: National Institute of Allergy and Infectious Diseases
NIH: National Institutes of Health
NO_3_: Nitrate
NTU: Nephelometric turbidity units
PCR: Polymerase chain reaction
PO_4_: Phosphate
Pr: Probe
R: Reverse
R2A: Reasoner’s 2A agar
RCF: Relative centrifugal force
SD: Standard deviation
sDW: Sterilized drinking water
sWW: Sterilized well water
TNTC: Too numerous to count
TOC: Total organic carbon
USEPA: United States Environmental Protection Agency
