# Supplementary material for: Early detection of viable Francisella tularensis in environmental matrices by culture-based PCR
Source: BMC Microbiol. 2020 Mar 25;20:66. doi: 10.1186/s12866-020-01748-0 (PMC7093956; doi:10.1186/s12866-020-01748-0)
Supplement: Supplementary file 1 — Additional file 1: SFigure. Control wells containing no live F. tularensis in the presence of inactivated target cells. Wells containing no live IN99 (A) or Schu4 (B) cells but 0 (circle), 102 (square), 104 (triangle), or 106 (diamond) of the respective isopropanol inactivated target cells confirmed that treated cells were not viable and that no change in PCR signal was observed over time. Data are from three replicates and presented as mean ΔCT values with error bars indicating standard deviation. [file 12866_2020_1748_MOESM1_ESM.pdf]

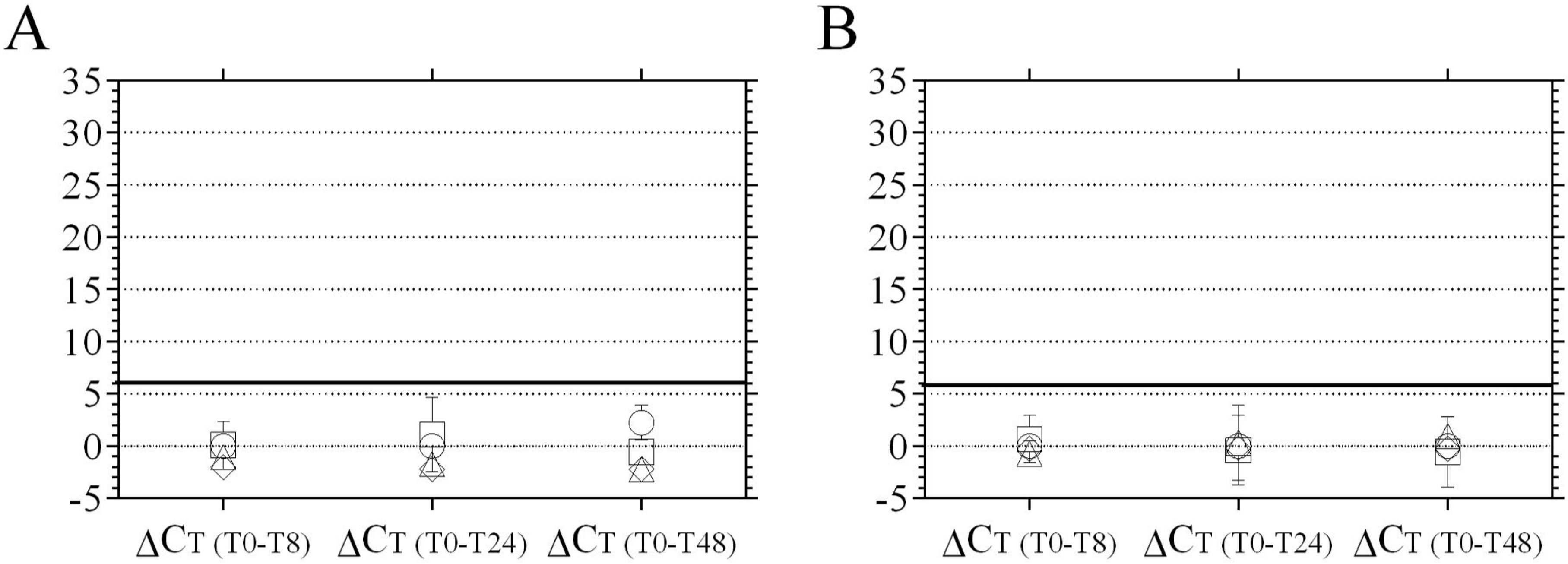

Supplemental Figure. Control wells containing no live *F. tularensis* in the presence of inactivated target cells. Wells containing no live IN99 (A) or Schu4 (B) cells but 0 (circle), 10^2 (square), 10^4 (triangle), or 10^6 (diamond) of the respective isopropanol inactivated target cells confirmed that treated cells were not viable and that no change in PCR signal was observed over time. Data are from three replicates and presented as mean ΔC_T values with error bars indicating standard deviation.
